# Dynamic alteration of poroelastic attributes as determinant membrane nanorheology for endocytosis of organ specific targeted gold nanoparticles

**DOI:** 10.1186/s12951-022-01276-1

**Published:** 2022-02-08

**Authors:** Tanmay Kulkarni, Debabrata Mukhopadhyay, Santanu Bhattacharya

**Affiliations:** 1 Department of Biochemistry and Molecular Biology, Mayo College of Medicine and Science, Griffin 413, Mayo Clinic Florida, 4500 San Pablo Road S, Jacksonville, FL 32224 USA; 2 Department of Physiology and Biomedical Engineering, Mayo College of Medicine and Science, Jacksonville, FL USA

**Keywords:** Poroelasticity, Targeted gold nanoparticle, Pancreatic cancer, Receptor mediated endocytosis, Plectin-1 targeting, Drained Poisson’s ratio, Effective shear stress, Diffusion coefficient, Pore size and Real-time membrane dynamics

## Abstract

**Background:**

Efficacy of targeted drug delivery using nanoparticles relies on several factors including the uptake mechanisms such as phagocytosis, macropinocytosis, micropinocytosis and receptor mediated endocytosis. These mechanisms have been studied with respect to the alteration in signaling mechanisms, cellular morphology, and linear nanomechanical properties (NMPs). Commonly employed classical contact mechanics models to address cellular NMPs fail to address mesh like structure consisting of bilayer lipids and proteins of cell membrane. To overcome this technical challenge, we employed poroelastic model which accounts for the biphasic nature of cells including their porous behavior exhibiting both solid like (fluid storage) and liquid like (fluid dissipate) behavior.

**Results:**

In this study, we employed atomic force microscopy to monitor the influence of surface engineering of gold nanoparticles (GNPs) to the alteration of nonlinear NMPs such as drained Poisson’s ratio, effective shear stress, diffusion constant and pore dimensions of cell membranes during their uptake. Herein, we used pancreatic cancer (PDAC) cell lines including Panc1, AsPC-1 and endothelial cell (HUVECs) to understand the receptor-dependent and -independent endocytosis of two different GNPs derived using plectin-1 targeting peptide (PTP-GNP) and corresponding scrambled peptide (sPEP-GNP). Compared to untreated cells, in case of receptor dependent endocytosis of PTP-GNPs diffusion coefficient altered ~ 1264-fold and ~ 1530-fold and pore size altered ~ 320-fold and ~ 260-fold in Panc1 and AsPC-1 cells, respectively. Whereas for receptor independent mechanisms, we observed modest alteration in diffusion coefficient and pore size, in these cells compared to untreated cells. Effective shear stress corresponding to 7.38 ± 0.15 kPa and 20.49 ± 0.39 kPa in PTP-GNP treatment in Panc1 and AsPC-1, respectively was significantly more than that for sPEP-GNP. These results demonstrate that with temporal recruitment of plectin-1 during receptor mediated endocytosis affects the poroelastic attributes of the membrane.

**Conclusion:**

This study confirms that nonlinear NMPs of cell membrane are directly associated with the uptake mechanism of nanoparticles and can provide promising insights of the nature of endocytosis mechanism involved for organ specific drug delivery using nanoparticles. Hence, nanomechanical analysis of cell membrane using this noninvasive, label-free and live-cell analytical tool can therefore be instrumental to evaluate therapeutic benefit of nanoformulations.

**Graphical Abstract:**

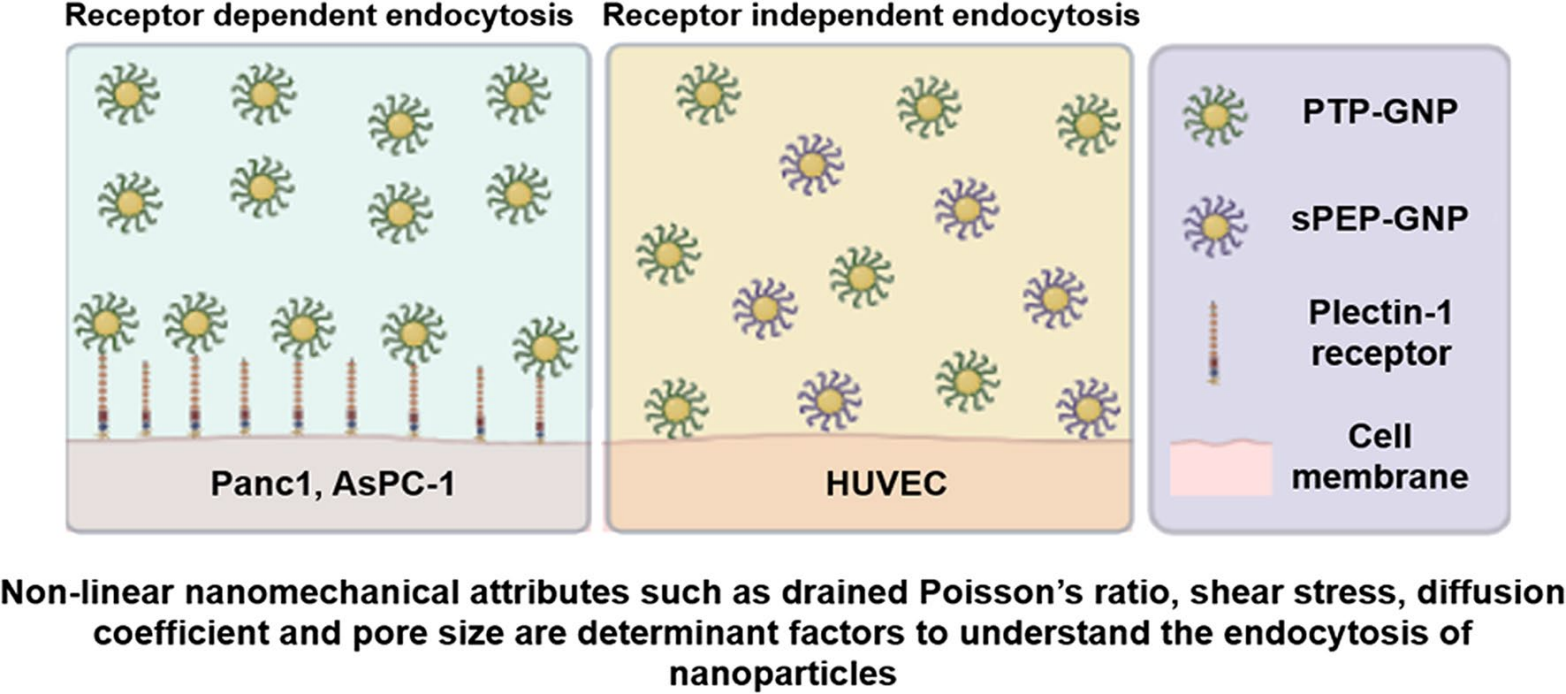

**Supplementary Information:**

The online version contains supplementary material available at 10.1186/s12951-022-01276-1.

## Background

Nanoparticles have gained significant attention due to its wide array of applications ranging from food, electronic to engineering and biomedical industry [1–3]. Synthesis of nanoparticles and their application in targeted drug delivery has become a prime focus in cancer treatment to improve its therapeutic outcome [4–11]. Extensive research has been documented to evaluate the efficacy of targeted drug delivery using gold nanoparticles (GNPs), and identify their uptake mechanisms such as phagocytosis, macropinocytosis, micropinocytosis and receptor mediated endocytosis [12–15]. Not only in cancer but also in endothelial biology, it is very crucial to evaluate the uptake of drug conjugated nanoparticles by the blood brain barrier (BBB) that can effectively cross the latter with minimal to no side effects [16]. These along with mathematical modeling and simulation studies have enabled the researchers to achieve significant strides in comprehending alteration in signaling mechanisms, cellular morphology and linear nanomechanical properties (NMPs) that include membrane stiffness, deformation and adhesion using atomic force microscope (AFM) [17–22]. Cell is largely composed of cytosolic fluid and is susceptible to both external and internal biomechanical stimuli, which triggers signaling pathways such as intracellular signal transduction, PI3K/AKT and kinase signalling that corresponds to the cell proliferation, differentiation and apoptosis [17, 18]. For instance, in cardiovascular system, physical stimuli can be sensed by the cells and in turn transmitted through intracellular signal transduction pathways to the nucleus, thus resulting in cell apoptosis [18]. Fibronectin 1, one of the crucial extracellular matrix (ECM) components affects cellular proliferation and apoptosis of human Glioma cells through PI3K/AKT signaling pathway [23]. Lastly, the role of mechanical stretch in fetal lung cells (E19 type II cells) was studied and the group observed that cellular differentiation occurred via an epidermal growth factor receptor-extracellular regulated protein kinase signaling pathway [24]. On the frontline of nanomechanical studies, cell adhesion mechanism was explored from the force measurements acquired at the multiple-bond level using leukocyte function associated antigen-1 (LFA-1)/intercellular adhesion molecule-1 (ICAM-1) as a model system [25]. Another comprehensive cell membrane stiffness study evaluated in human mammary epithelial cells in various cell cycle as well as microenvironments related to cell–cell contacts led to the findings that these cells become softer as they advance to the tumorigenic phase and then stiffens in their progression to metastatic cells [26]. AFM has also been employed to study the endocytosis in which, the mesh like structure of cellular membrane enables it to interact with the surrounding via exchange of cytosolic fluids as well as allowing uptake of external moieties such as nanoparticles through various endocytosis mechanisms [27, 28]. During this exchange process, cellular cytoskeleton undergoes series of dynamic alterations affecting overall cellular rheology and in turn affecting its NMPs such as membrane stiffness, deformation and adhesion [29–31]. However, alteration in poroelasticity parameters during various endocytosis mechanisms remains to be studied and could potentially yield a more in-depth perception that will boost our understanding of endocytosis mechanisms.

Several classical contact mechanics model have been incorporated to study cellular rheology such as Hertz and Sneddon model [32, 33]. However, these models neglect the cellular adhesion with the probe, thus failing to do the desired justice. To address this, in case of larger diameter (micron range) probes, Derjaguin-Muller-Toporov and Johnson–Kendall–Roberts model are commonly incorporated that account for the thermodynamic work of adhesion [34–36]. In addition, power law model is also employed to study viscoelasticity of cells as they tend to be viscous [37–40]. However, these models fall short as they fail to address the biphasic nature of cell’s cytoplasm [41]. Poroelasticity model aids in studying the biphasic cytoplasm consisting of porous elastic mesh comprising of cytoskeleton, organelles and macromolecules amidst interstitial cytosolic fluid [42–45]. Poroelasticity model acknowledges that the response of cell membrane to an external stimulus is both time and length scale dependent [46–48]. Poroelasticity model enables quantification of cellular rheology in terms of drained Poisson’s ratio, diffusion coefficient and pore size, which allows exploring and correlating them with various biological phenomena [41, 44, 49, 50]. Another study focussed on poroelastic behavior of cells using a micron size bead demonstrating that cytoskeletal components such as actin, microtubules intermediate filaments and myosin affected the diffusion coefficient of cell [41]. In another study, alterations in poroelastic parameters of hepatocellular carcinoma (SMMC-7721) cells treated with fullerenol were explored [49]. Fullerenol treated cells exhibited a significant increase in the pore size and a slight decrease in elastic modulus [49], providing insights into cancer therapies. Thus, poroelastic model which accounts for the biphasic nature of cells including their porous behavior exhibiting both solid like (fluid storage) and liquid like (fluid dissipate) behavior could prove detrimental to our endocytosis understanding.

Previously, we successfully demonstrated synthesis of plectin-1 targeting peptide (PTP) conjugated with gold nanoparticles followed by its comprehensive characterization in terms of particle size, drug conjugation and architecture of the peptide-grafted nanoassembly both experimentally and via simulation. These GNPs demonstrated higher cytotoxicity in vitro in Panc1 and AsPC1 cell lines as well as antitumor efficacy in vivo in Panc1 orthotopic xenograft model through selective uptake in pancreatic ductal adenocarcinoma (PDAC) tumor tissues [4]. Recently, using these GNPs we demonstrated dynamic alterations in linear NMPs using AFM to understand the uptake mechanisms and cell membrane associated nanomechanics. We observed softening of cell membrane in receptor dependent endocytosis as opposed to receptor independent endocytosis in which, the membrane appeared to become stiffer [12]. We also demonstrated direct correlation between membrane stiffness and surface plectin-1 receptors resulting from PTP-GNP uptake by the PDAC cells. In this work, we explore the dynamic alterations in poroelastic parameters of PDAC cell lines (Panc1 and AsPC-1 cells) when treated with PTP-GNP (receptor dependent). We further compare this behavior with human umbilical vein endothelial cells (HUVECs) treated with PTP-GNP as well as scrambled peptide conjugated GNP (sPEP-GNP) and Panc1 and AsPC-1 treated with sPEP-GNP, all exhibiting receptor independent endocytosis mechanisms. This poroelasticity study will open new avenues into comprehending dynamic changes in the cell membrane architecture over a broader time scale such as in endocytosis mechanisms that are temporal dependent. Insights into nonlinear poroelastic parameters during receptor dependent and independent endocytosis mechanisms from this study, will aid in understanding cellular behavior during therapeutic targeting.

## Results

### AFM tool to evaluate time dependent poroelastic signatures from Force-relaxation curves

**Fig. 1:**
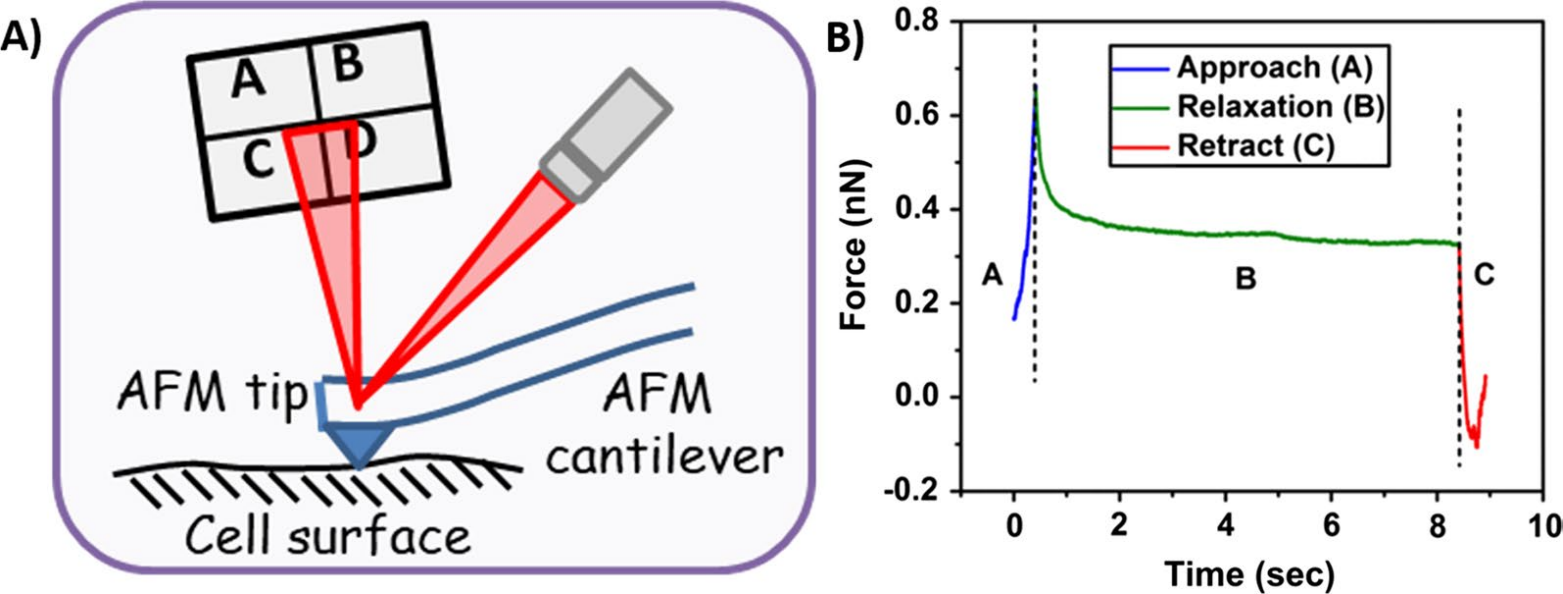
Experimental setup for Force-relaxation (F-R). **A** Schematic of AFM tip probing cell surface to acquire an F-R curve. **B** A representative F-R curve exhibiting approach, relaxation and retraction steps to constitute an F-R curve

Cells exhibiting poroelastic behavior has been well-established [41, 44, 49, 50]. Several prior studies have indicated severe heterogeneity in cell surface nanomechanics [51, 52]. In addition, irregularities exist in their nanomechanical signatures due to varying cell cycle progression [51]. To overcome these discrepancies, we arrested Panc1, AsPC-1 and HUVEC cells in S-phase and performed ramp scripting on a narrow (500 × 50 nm^2^) region over the nuclear membrane as shown in Fig. 1A experimental schematic. Arresting cells in particular cell cycle phase yields homogenous cellular population of cells with uniform NMPs [53]. Force-relaxation (F-R) curve is often employed on the cell surface to study the time and length scale alterations in force during the tip-sample interactions [50]. In nanoindentation procedure that yields force separation curve, a typical interaction of the probe with sample surface lasts for a few milliseconds and the probe begins its retraction cycle immediately after attaining the applied triggered force. However, in ramp script procedure to study the time and length scale poroelastic properties, a tip has to remain in contact with the sample surface for a longer duration (typically a few seconds) giving the relaxation in the force once the indentation reaches a predefined value.

In this study, we initially performed nanoindentation procedure to determine the force required to achieve a 10% indentation corresponding to the overall cellular height for Panc1, AsPC-1 and HUVEC. This force was then used in ramp script studies to achieve and maintain indentation of 400 nm, 350 nm and 200 nm for Panc1, AsPC-1 and HUVECs, respectively. We observed that same force level was able to achieve variable deformation values specific to cell lines and can be attributed to their intrinsic membrane stiffness in the absence of any treatment. A representative F-R curve is shown in Fig. 1B. It comprises of three regions viz. approach, relaxation and retraction. During the approach segment, the cantilever moves towards the sample at high forward velocity until the desired indentation is achieved. During the approach process, the cytosolic fluid gets trapped inside the cellular membrane. Following which, relaxation in force was monitored over a period of 8 s by maintaining the indentation depth. During the relaxation period, force begins to drop exponentially and ultimately reaches a plateau indicating that the redistribution of interstitial fluid and the force imposed by the tip is balanced by the stress in the elastic porous matrix. Final segment of F-R curve is the retract curve during which, the probe overcomes attractive pull-off force exerted by the sample. In order to evaluate poroelasticity parameters, relaxation (middle segment) was analyzed using MATLAB programming software.

### Force-relaxation (F-R) curves

**Fig. 2:**
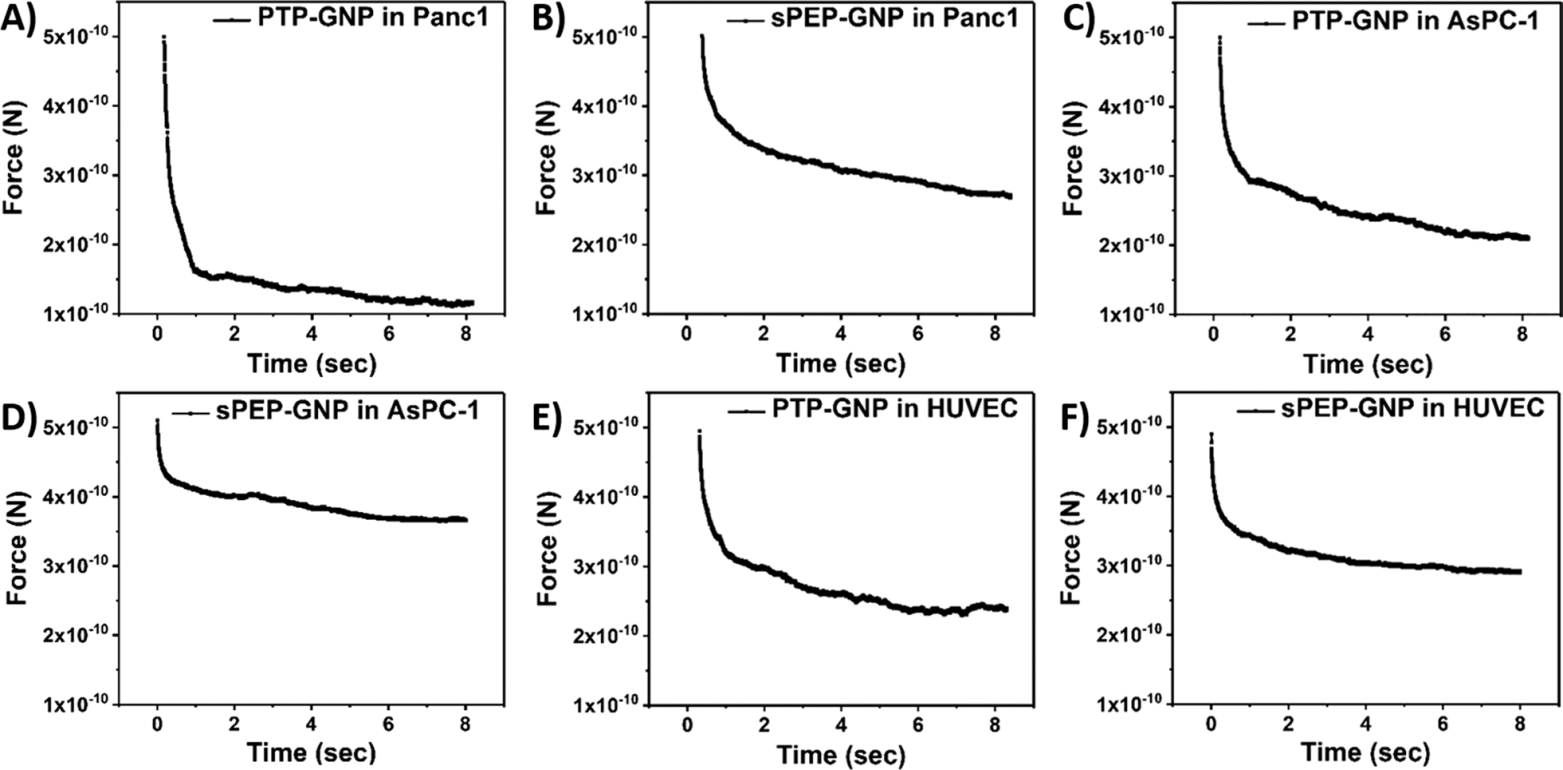
Representative relaxation segments from force-relaxation curves for various GNP formulation treatments in different cell lines. **A** PTP-GNP in Panc1. **B** sPEP-GNP in Panc1. **C** PTP-GNP in AsPC-1. **D** PTP-GNP in HUVEC. **E** sPEP-GNP in HUVEC. **F** sPEP-GNP in AsPC-1

Intracellular movements of small molecules and large aggregates occur over a broad time scale. Due to which, we only consider the middle segment exhibiting relaxation in force levels at constant indentation over a larger time scale as shown in Fig. 2 for various treatments in different cell lines. Here, F-R curve corresponding to PTP-GNP treatment in Panc1 and AsPC-1 cells exhibit receptor dependent endocytosis phenomena as shown in Fig. 2A and C. However, other treatments such as PTP-GNP and sPEP-GNP in HUVECs as well as sPEP-GNP in Panc1 and AsPC-1 cells represent receptor independent endocytosis mechanisms as shown in Fig. 2B and D–F.

Before performing F-R curves, nanoindentation experiment was performed on all three cell lines to achieve a force that resulted in less than 10% deformation. We observed that a force of 500 pN was sufficient to maintain the 10% criteria that avoided permanent deformations in the cell lines. F-R curves during receptor dependent endocytosis process presented a steeper slope compared to the receptor independent endocytosis scenario. As these curves represent a decayed exponential curve with two distinct slopes, curve fitting is performed on these curves using Eq. 6 to evaluate characteristic decay time. However, these F-R curves are only qualitative measure of alterations in nonlinear poroelasticity parameters based on slope alterations for respective treatments. Clear distinction in the slope between the F-R curves for various treatments and no treatment is evident from Fig. 2 and Addition file 1: Figure S1 for respective cell lines. To quantify poroelasticity parameters, we used a series of mathematical equations presented above Eqs. 1–8 and evaluated diffusion coefficient and pore size.

### Dynamic alterations in drained Poisson’s ratio during receptor dependent and independent endocytosis mechanisms

As endocytosis is a dynamic process, it becomes crucial to monitor time dependent alterations in cellular nanomechanics. Plectin-1 is identified as a receptor explicitly overexpressed on pancreatic cancer cell surface and has been extensively explored as a potential biomarker since its advent [54]. In addition, our recent work exhibits the overexpression of plectin-1 in both primary and immortalized pancreatic cancer cell lines including Panc1 and AsPC-1 [12]. Plectin-1 targeted peptide from PTP-GNP binds to the plectin-1 surface receptors in Panc1 cells and internalizes leading to a receptor dependent endocytosis mechanism. Scrambled peptide bound GNP (sPEP-GNP) on the other hand internalizes via receptor independent mechanisms in Panc1 cells as there is no favorable binding complex to the plectin-1 surface receptors. In addition, HUVECs do not possess surface plectin-1 receptors [12], hence the internalization of PTP-GNP and sPEP-GNP occur via receptor independent endocytosis mechanism. To confirm and validate our findings pertaining to alteration in poroelasticity parameters during receptor dependent and independent endocytosis mechanisms, we chose another PDAC cell line, AsPC-1 that is known to exhibit surface plectin-1 receptors [12]. PTP-GNP and sPEP-GNP internalization in AsPC-1 cells occur via receptor dependent and independent endocytosis mechanism, respectively. We first monitored whether the experimental parameters influence the poroelastic parameters in S phase arrested Panc1 cells over a 20-min time window, when probed over the same region in the increments of 5-min.

**Fig. 3:**
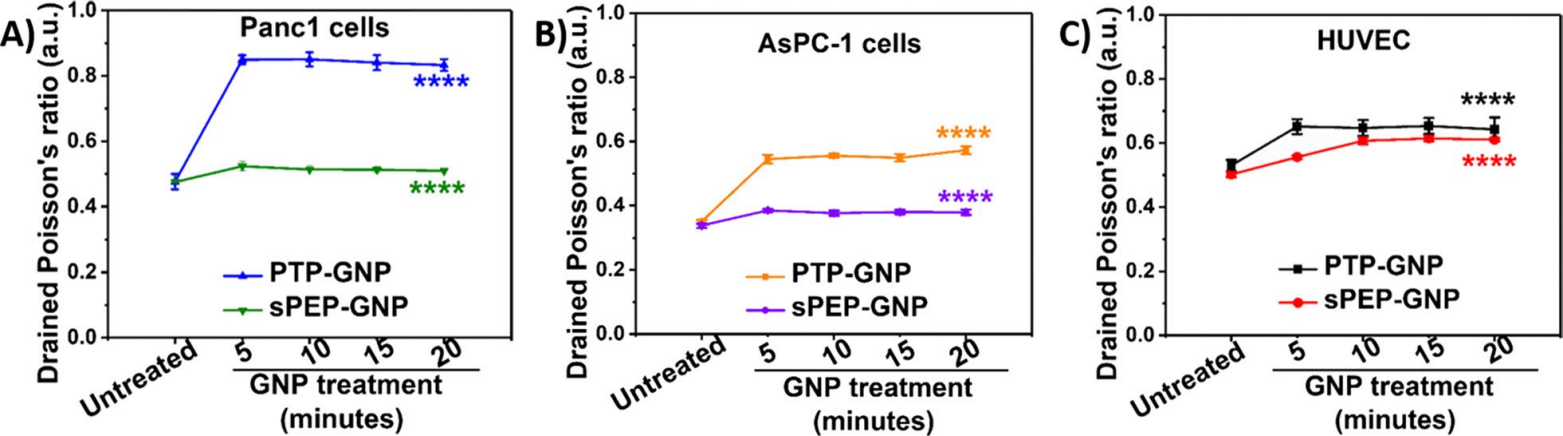
Temporal alteration in drained Poisson’s ratio. **A** In Panc1 cells during receptor dependent (PTP-GNP in Panc1) and receptor independent (sPEP-GNP in Panc1) endocytosis processes. **B** In AsPC-1 cells during receptor dependent (PTP-GNP in AsPC-1) and receptor independent (sPEP-GNP in AsPC-1) endocytosis processes. **C** In HUVECs with PTP-GNP and sPEP-GNP treatment both resembling receptor independent endocytosis process. (Statistical significance norm is as follows: ****, p < 0.0001.)

We did not observe any significant alterations in all three poroelastic attributes including drained Poisson’s ratio determined from Eq. 5, diffusion coefficient and pore size as seen from Addition file 1: Figure S2a–c. In our previous study, we demonstrated that NMPs of cells are significantly governed by the cell phase. As a result, cell synchronization is essential to overcome the heterogeneity in mechanical properties [51]. We verified whether the finding holds true in AsPC-1 cells as well by synchronizing the cells in S phase and comparing their poroelasticity values with asynchronous AsPC-1 cells as shown in Addition file 1: Figure S3a–c. We observed that while the individual parameters values remain consistent, there is a huge variation in poroelasticity parameters in asynchronous AsPC-1 cells as seen from Addition file 1: Figure S3a–c. We attribute this inconsistency to the asynchronous nature of AsPC-1 cells. For all the experiments involving AsPC-1 cells, we chose S phase synchronous AsPC-1 cells. And lastly, we evaluated poroelasticity properties of serum starved HUVECs and S phase Panc1 as shown in Addition file 1: Figure S4a–c. Except for drained Poisson’s ratio, other poroelasticity parameters exhibited non-significant difference. Differences in drained Poisson’s ratio could be attributed to the intrinsic cellular property. After establishing the baselines for all three cell lines and ensuring that the experimental trigger force does not cause permanent deformations in these cell lines, we monitored dynamic alterations in drained Poisson’s ratio of Panc1, AsPC-1 and HUVECs when treated with PTP-GNP and sPEP-GNP over a period of 20 min in the increments of 5 min as shown in Fig. 3A–C from Eq. 5. Untreated Panc1 cells exhibited a drained Poisson’s ratio of 0.47 ± 0.02 a.u. whereas, untreated AsPC-1 cells displayed lower drained Poisson’s ratio (0.34 ± 0.004 a.u.) compared to the untreated Panc1 cells. Untreated HUVECs exhibited a drained Poisson’s ratio of 0.52 ± 0.01 a.u. as seen from Fig. 3A–C. Upon PTP-GNP treatment in Panc1 cells and AsPC-1 cells, we observed an immediate increase of 1.8-fold and 1.6-fold in drained Poisson’s ratio in the initial 5 min (drained Poisson’s ratio of 0.85 ± 0.02 a.u. in Panc1 and 0.54 ± 0.01 a.u. in AsPC-1 cells, respectively), thereafter remaining more or less constant over the 20-min time window as seen from Fig. 3A and B. Although we observed an increase in drained Poisson’s ratio for receptor independent endocytosis, peak increase in drained Poisson’s ratio was observed at various time points for various cells with treatments. For instance, drained Poisson’s ratio for PTP-GNP in HUVEC (drained Poisson’s ratio of 0.65 ± 0.02 a.u.) and sPEP-GNP in Panc1 (drained Poisson’s ratio of 0.52 ± 0.01 a.u.) both reached a peak at 5-min timepoint, thereafter remaining more or less constant over the 20 min time window as shown in Fig. 3A and C. Interestingly, in the case of sPEP-GNP treatment in HUVEC, peak increase in drained Poisson’s ratio was observed at 15-min time point (drained Poisson’s ratio of 0.61 ± 0.008 a.u.), thereafter remaining constant for the rest of the 20-min time window. Similarly, we observed that when AsPC-1 cells were treated with sPEP-GNP, the treatment caused the drained Poisson’s ratio to increase to 0.38 ± 0.005 a.u. at the 5-min time point and remain consistent for the remaining duration as seen from Fig. 3B. Such behavior in receptor dependent and independent endocytosis mechanism in AsPC-1 cell line was consistent with Fig. 3A and C. Although an increase in drained Poisson’s ratio was observed for both receptor dependent and independent scenarios, fold change increase in receptor dependent endocytosis case was substantial compared to receptor independent as evident from Fig. 3A–C.

### Dynamic alterations in shear stress during receptor dependent and independent endocytosis mechanisms

The AFM tip rapidly indenting into the cell membrane surface, exerts a force perpendicular to the cell membrane. As a result, a shear force component arises parallel to the cellular cross-section [55]. Classically, shear stress is correlated to the cell membrane stiffness via material's intrinsic property as shown in Eq. 2. However, poroelasticity model yields two different types of shear stress such as undrained shear stress resulting from rapid nanoindentation Eq. 3 and drained shear stress resulting from the relaxation in force Eq. 4. The difference between the drained and undrained shear stress is defined as effective shear stress. While membrane stiffness is the ability of the cell membrane to withstand the applied force without any permanent fracture, shear stress is the ability of the cell membrane to resist the interstitial pressure created by the rapid indentation by the tip before the advent of the relaxation phase as shown in Fig. 1B. Both these properties are distinct nanomechanical attributes acquired from distinct experimental procedures. Here, we monitored the alterations in shear stress during these endocytosis mechanisms and further evaluate the effective change in the shear stress between the drained and undrained scenario from Eqs. 3 and 4.

**Fig. 4:**
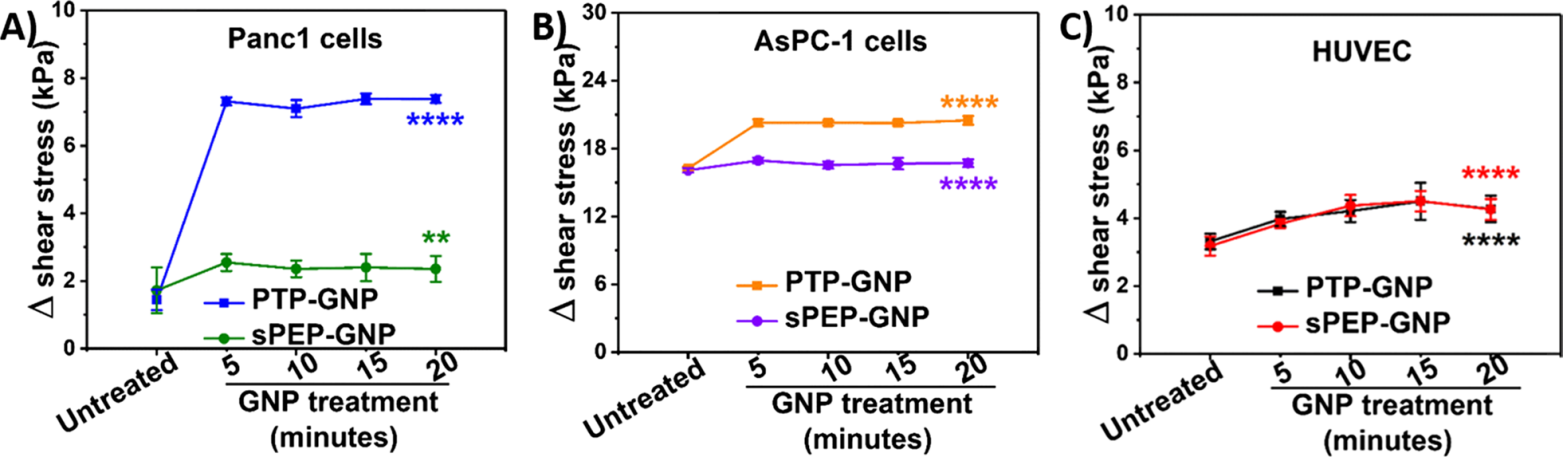
Temporal alteration in effective change in shear stress during the transition from undrained to drained state. **A** In Panc1 cells during receptor dependent (PTP-GNP in Panc1) and receptor independent (sPEP-GNP in Panc1) endocytosis processes. **B** In AsPC-1 cells during receptor dependent (PTP-GNP in AsPC-1) and receptor independent (sPEP-GNP in AsPC-1) endocytosis processes. **C** In HUVECs with PTP-GNP and sPEP-GNP treatment both resembling receptor independent endocytosis process. (Statistical significance norm is as follows: **, p < 0.01; ****, p < 0.0001.)

In the undrained case, shear stress is dictated by indentation, which varies for cell lines. The applied force and the tip radius are constant parameters. We observed that AsPC-1 cell lines exhibited maximum shear stress during the undrained state with 24.1 kPa, whereas HUVEC and Panc1 cell lines exhibited 10.4 kPa and 8.52 kPa shear stress, respectively determined from Eq. 3. After the built-up interstitial pressure during rapid indentation was dissipated (drained state), we observed that the shear stress was significantly lower than the undrained state. Different cell lines in untreated state exhibited varying drained shear stress such as 7.167 ± 0.09 kPa, 6.94 ± 0.19 kPa and 7.94 ± 0.12 kPa for HUVECs, Panc1 and AsPC-1 cell lines, respectively as seen from Addition file 1: Figure S4a-S4c. For receptor dependent scenario (PTP-GNP in Panc1 and AsPC-1), we observed an immediate decrease in drained shear stress at 5-min time point with values corresponding to 1.21 ± 0.11 kPa and 3.83 ± 0.3 kPa, respectively compared to the untreated cells as shown in Addition file 1: Figure S5a and S5b. For PTP-GNP treatment in Panc1 and AsPC-1 cell lines, maximum drop in drained shear stress value was observed at 15-min time point and 20-min time point with value corresponding to 1.13 ± 0.15 kPa and 3.61 ± 0.38 kPa, respectively as evaluated from Eq. 4. Furthermore, for receptor independent scenario (PTP-GNP and sPEP-GNP in HUVECs, sPEP-GNP in Panc1 and AsPC-1), we observed a significantly lesser drop in drained shear stress compared to the receptor dependent case as seen from Addition file 1: Figure S5a-S5c. In the case of HUVECs, both GNP treatments induced a decreasing trend in drained shear stress, which was maximum at 15-min time point. With values corresponding to 5.91 ± 0.54 kPa and 5.91 ± 0.3 kPa for PTP-GNP and sPEP-GNP treatments, respectively as seen from Addition file 1: Figure S5c. For sPEP-GNP treatments in Panc1 and AsPC-1 cell lines, we observed a maximum decrease in drained shear stress corresponding to 15-min time point (Panc1 cells) and 5-min time point (AsPC-1 cells) with values corresponding to 5.9 ± 0.25 kPa and 7.15 ± 0.24 kPa, respectively as seen from Addition file 1: Figure S5a and S5b. In terms of effective change in shear stress transitioning from undrained to drained scenario for various cell lines, we observed that receptor dependent case exhibited maximum alteration with values corresponding to 7.38 ± 0.15 kPa and 20.49 ± 0.39 kPa for PTP-GNP treatment in Panc1 and AsPC-1 cell lines at 15-min and 20-min time point, respectively as seen from Fig. 4A and B. In the case of receptor independent endocytosis mechanism, we observed an effective change in shear stress for Panc1 and AsPC-1 cell lines treated with sPEP-GNP with values corresponding to 2.4 ± 0.4 kPa and 16.9 ± 0.24 kPa at 15-min and 5-min time point, respectively (Fig. 4A and B). For HUVECs treated with both PTP-GNP and sPEP-GNP (both resembling receptor independent endocytosis mechanisms), we observed an effective change in shear stress at 15-min time point with values corresponding to 4.5 ± 0.5 kPa and 4.5 ± 0.3 kPa, respectively as seen from Fig. 4C. Therefore, from these observations we conclude that the effective change in shear stress for receptor dependent endocytosis scenario was significantly more prominent than the receptor independent scenario for respective cell lines.

### Alterations in diffusion coefficient signature during receptor dependent and independent endocytosis mechanisms

**Fig. 5:**
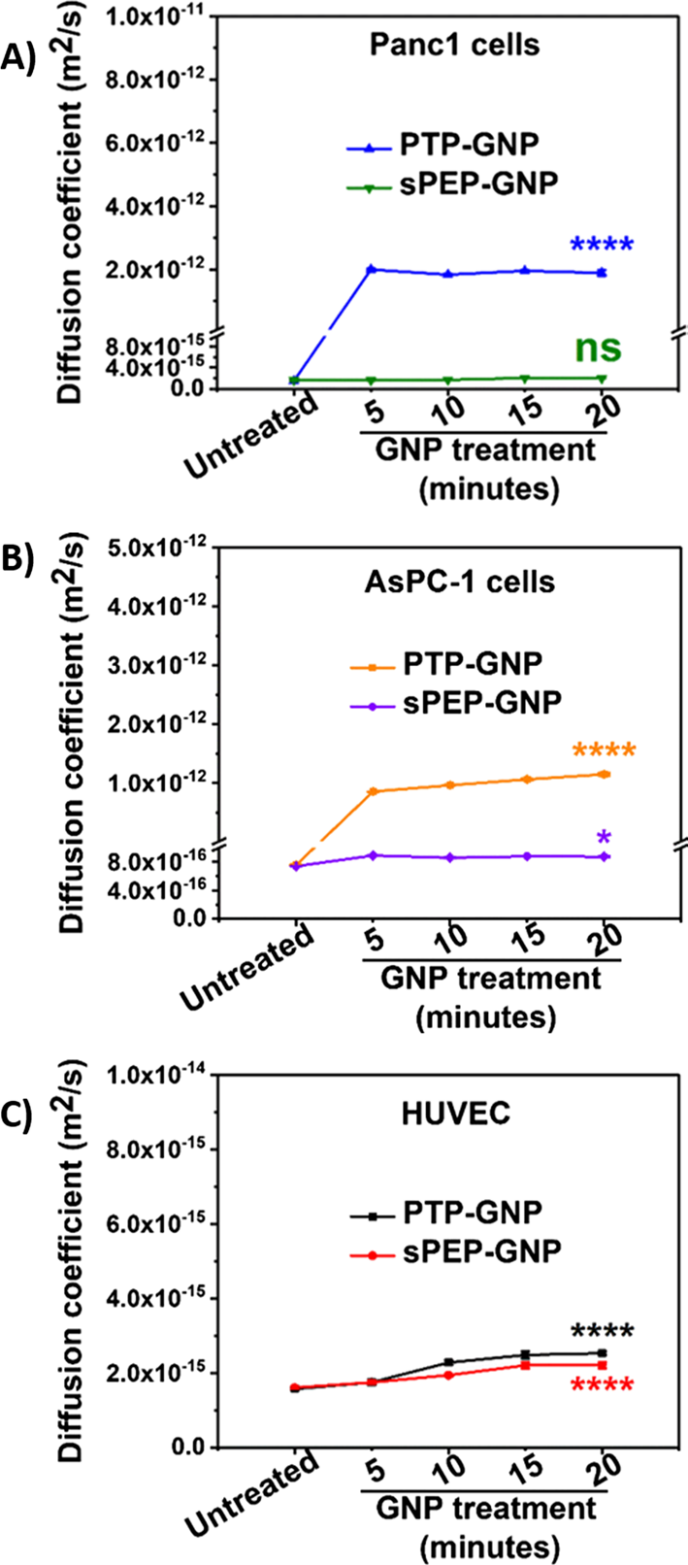
Temporal alteration in diffusion coefficient. **A** In Panc1 cells during receptor dependent (PTP-GNP in Panc1) and receptor independent (sPEP-GNP in Panc1) endocytosis processes. **B** In AsPC-1 cells during receptor dependent (PTP-GNP in AsPC-1) and receptor independent (sPEP-GNP in AsPC-1) endocytosis processes. **C** In HUVECs with PTP-GNP and sPEP-GNP treatment both resembling receptor independent endocytosis process. (Statistical significance norm is as follows: ns, not significant; *,p < 0.05; ****, p < 0.0001.)

We then monitored the diffusion coefficient for both receptor dependent and receptor independent endocytosis mechanisms in Panc1, HUVECs and AsPC-1 cell lines as shown in Fig. 5A–C. Diffusion coefficient is a measure of the diffusion of cytosolic fluid that is trapped inside the cell membrane due to the rapid pressure buildup during approach phase of F-R experiment. During the relaxation segment, this built-up pressure is released in the form of the cytosolic fluid diffusion causing a change in diffusion coefficient, which was evaluated using Eq. 7. Untreated Panc1 cells and AsPC-1 cells exhibited a diffusion coefficient of 1.58 × 10^–15^ ± 2 × 10^–16^ m^2^/s and 7.51 × 10^–16^ ± 4.3 × 10^–18^ m^2^/s, respectively, whereas HUVECs exhibited a diffusion coefficient of 1.59 × 10^–15^ ± 9.96 × 10^–17^ m^2^/s as seen from Fig. 5A–C. We observed that during the receptor dependent endocytosis mechanism (PTP-GNP in Panc1 and AsPC-1 cells), there occurred ~ 1264-fold change and ~ 1530-fold change in diffusion coefficient (2 × 10^–12^ ± 3.22 × 10^–14^ m^2^/s in Panc1 and 1.15 × 10^–12^ ± 2.28 × 10^–14^ m^2^/s in AsPC-1 cells) after the 5-minutetime point and 20-min time point for Panc1 and AsPC-1 cells, respectively as seen from Fig. 5A and B. Upon PTP-GNP treatment in AsPC-1 cells, we observed a systematic increase in the diffusion coefficient upto 20-min time window. Although we observed an increasing trend in diffusion coefficient, we maintained the 20-min time window for consistency. On the other hand, for receptor independent endocytosis mechanisms, a very minimal increase in diffusion coefficient (~ 1.1-fold) was observed for PTP-GNP in HUVEC (1.74 × 10^–15^ ± 7.4 × 10^–17^ m^2^/s) and sPEP-GNP in HUVEC (1.75 × 10^–15^ ± 4.84 × 10^–17^ m^2^/s) compared to the respective controls, as shown in Fig. 5C. No significant change in diffusion coefficient for sPEP-GNP treatment in Panc1 was observed during the 5-min time point. Thereafter, we observed a systematic increase for the next two time points for receptor independent endocytosis process. A fold-increase of 1.61, 1.38 and 1.27 was observed in the case of PTP-GNP treatment in HUVEC, sPEP-GNP in HUVEC and sPEP-GNP in Panc1, respectively as seen from Fig. 5A and C. For receptor independent endocytosis, we observed non-significant changes in diffusion coefficient post 5-min time point until 20 min.

We further observed the behavior of sPEP-GNP in AsPC-1 cells also resembling receptor independent endocytosis over the same time window as shown in Fig. 5B. When AsPC-1 cells were treated with sPEP-GNP, we observed an increase in the diffusion coefficient after 5 min of the treatment with value corresponding to 8.92 × 10^–16^ ± 2.03 × 10^–18^ m^2^/s, thereafter remaining constant over a 20-min time window. Such a behavior was consistent in receptor independent endocytosis mechanisms and associated change in diffusion coefficient in Panc1 and HUVECs with various treatments as seen from Fig. 5A–C. These results indicate the true dynamic nature of the endocytosis mechanism and diffusion coefficient could serve as an important characteristic to differentiate these endocytosis mechanisms.

### Alterations in pore size signature during receptor dependent and independent endocytosis mechanisms

**Fig. 6:**
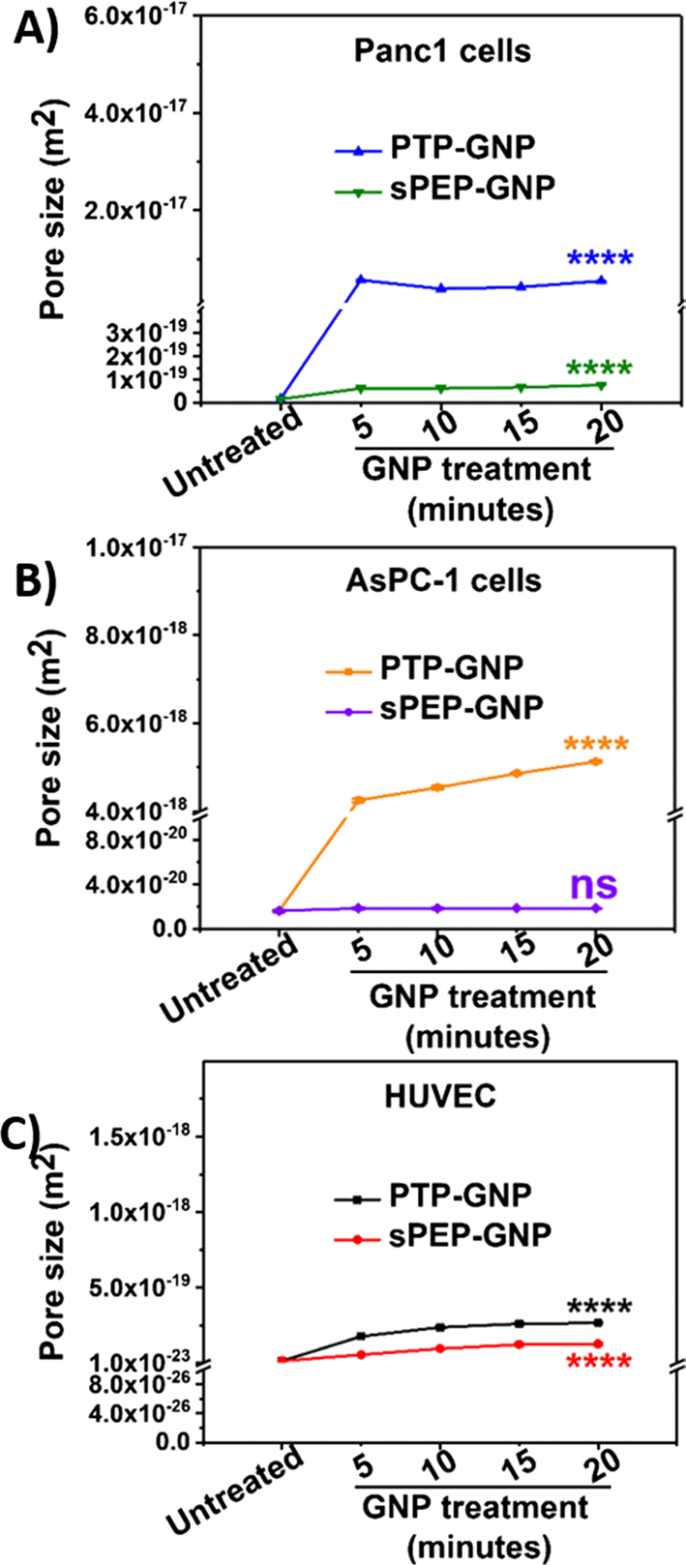
Temporal alteration in pore size. **A** In Panc1 cells during receptor dependent (PTP-GNP in Panc1) and receptor independent (sPEP-GNP in Panc1) endocytosis processes. **B** In AsPC-1 cells during receptor dependent (PTP-GNP in AsPC-1) and receptor independent (sPEP-GNP in AsPC-1) endocytosis processes. **C** In HUVECs with PTP-GNP and sPEP-GNP treatment both resembling receptor independent endocytosis process. (Statistical significance norm is as follows: ns, not significant; ****, p < 0.0001.)

We further probed into the alterations associated with the pore size factor with both receptor dependent and independent endocytosis mechanisms, that were evaluated using Eq. 8. Consistent with the observed trends in the case of drained Poisson’s ratio and diffusion coefficient, pore size too altered drastically for receptor dependent endocytosis mechanism as shown in Fig. 6A and B. Untreated Panc1, AsPC-1 and HUVECs possessed a pore size of 1.76 × 10^–20^ ± 3.02 × 10^–22^ m^2^ 1.65 × 10^–20^ ± 3.1 × 10^–22^ m^2^ and 1.41 × 10^–20^ ± 1.17 × 10^–22^ m^2^, respectively as seen from Fig. 6A–C. A fold increase of ~ 320 (5.69 × 10^–18^ ± 9.29 × 10^–20^ m^2^) was observed during the initial 5-min time window for PTP-GNP treatment in Panc1 as seen from Fig. 6A. Thereafter, a sharp drop in pore size was observed, (~ 100-fold decrease compared to the 5^th^ minute time point) following which we observed a systematic increase as seen from Fig. 6A. This inconsistent trend for pore size in receptor dependent endocytosis mechanism could be attributed to the dynamic endocytosis mechanism during which, surface plectin-1 receptor expression levels are constantly changing due to the internalization leading to pore size variability. At the 20^th^ minute time point, it regained its fold increase to that of 5^th^ minute time point. When AsPC-1 cells were treated with PTP-GNP, we observed ~ 250-fold increase in pore size at the end of 5 min (4.25 × 10^–18^ ± 3.88 × 10^–20^ m^2^) compared to untreated AsPC-1 cells as seen from Fig. 6B. Here, we observed a systematic increasing trend in pore size until the 20-min time window, where peak pore size of 5.13 × 10^–18^ ± 2.65 × 10^–20^ m^2^ was observed, which was different from PTP-GNP treatment in Panc1 cells.

Within receptor independent scenarios, the rate of increase in pore size differed as evident from the slope changes. PTP-GNP in HUVECs showed maximum fold change at the end of 20^th^ minute time window (2.65 × 10^–19^ ± 5.94 × 10^–21^ m^2^) exhibiting ~ 18-fold increase compared untreated HUVECs, whereas sPEP-GNP in HUVEC exhibited a pore size of 1.26 × 10^–19^ ± 7.56 × 10^–21^ m^2^ (~ 9.8-fold increase) and sPEP-GNP in panc1 displayed a pore size of 7.75 × 10^–20^ ± 5.04 × 10^–22^ m^2^ (~ 4.4-fold increase) as shown in Fig. 6A and C. Similar to the other poroelastic parameters mentioned before, we confirmed the changes in pore size during receptor independent endocytosis mechanisms using AsPC-1 cell line and maintained consistency in experimental parameters and time window.. The receptor independent endocytosis mechanism scenario exhibited by the sPEP-GNP treated AsPC-1 cells did not show a significant increase in the pore size over the 20-min time window. The maximum increase was observed at 5th minute, corresponding to the pore size value of 1.88 × 10^–20^ ± 1.28 × 10^–22^ m^2^ as seen from Fig. 6B. These results confirm the dynamic nature of the endocytosis mechanisms and the alterations in the cellular rheology introduced by the endocytosis. The pore size can be further explored to form one of the key signatures along with diffusion coefficient to distinguish various endocytosis mechanisms.

## Discussion

Surface modified GNPs are extensively researched and optimized for effective cancer therapy [6, 10, 15]. Previously, we have shown uptake of PTP-GNP and sPEP-GNP in PDAC in vitro and in vivo system [4, 12]. During the cellular uptake, cellular cytoskeleton undergo alteration to facilitate the endocytosis of nanoparticles. Several studies have reported that the components of cellular cytoskeleton such as actin filaments, microtubules, intermediate keratin filaments and myosin play a pivotal role in regulating cellular NMPs [30, 56–59]. However, these studies along with our recently published studies focused on liner nanomechanical characteristics such as membrane stiffness, adhesion and deformation [12]. Typical soft samples such as cells possess sponge-like porous elastic matrix through which exchange of interstitial fluid as well as nanoparticles takes place via various endocytosis mechanisms [60, 61]. Depending on the endocytosis mechanisms, the cell membrane undergoes dynamic alterations, which in turn affect their mechanical properties [12]. Also, as endocytosis process is dynamic in nature, it becomes vital to study the temporal dynamicity in nanomechanical alterations at the cell membrane [12, 62]. Intracellular exchange of small molecules and large aggregates occur over a broad time scales [26, 63]. A conventional nanoindentation experiment that typically occurs over a few millisecond periods yields instantaneous linear NMPs. Whereas force-relaxation (F-R) measurements shed further insights into cellular nanomechanics in terms of poroelasticity parameters better suited for such broad time scale processes. In the field of AFM, it is immensely vital to maintain the deformation within 10% of the sample height to prohibit excess prodding that might result into permanent deformation in soft samples such as cells. In our study, we have optimized the applied force such that the overall deformation falls within the above-mentioned criteria.

**Fig. 7:**
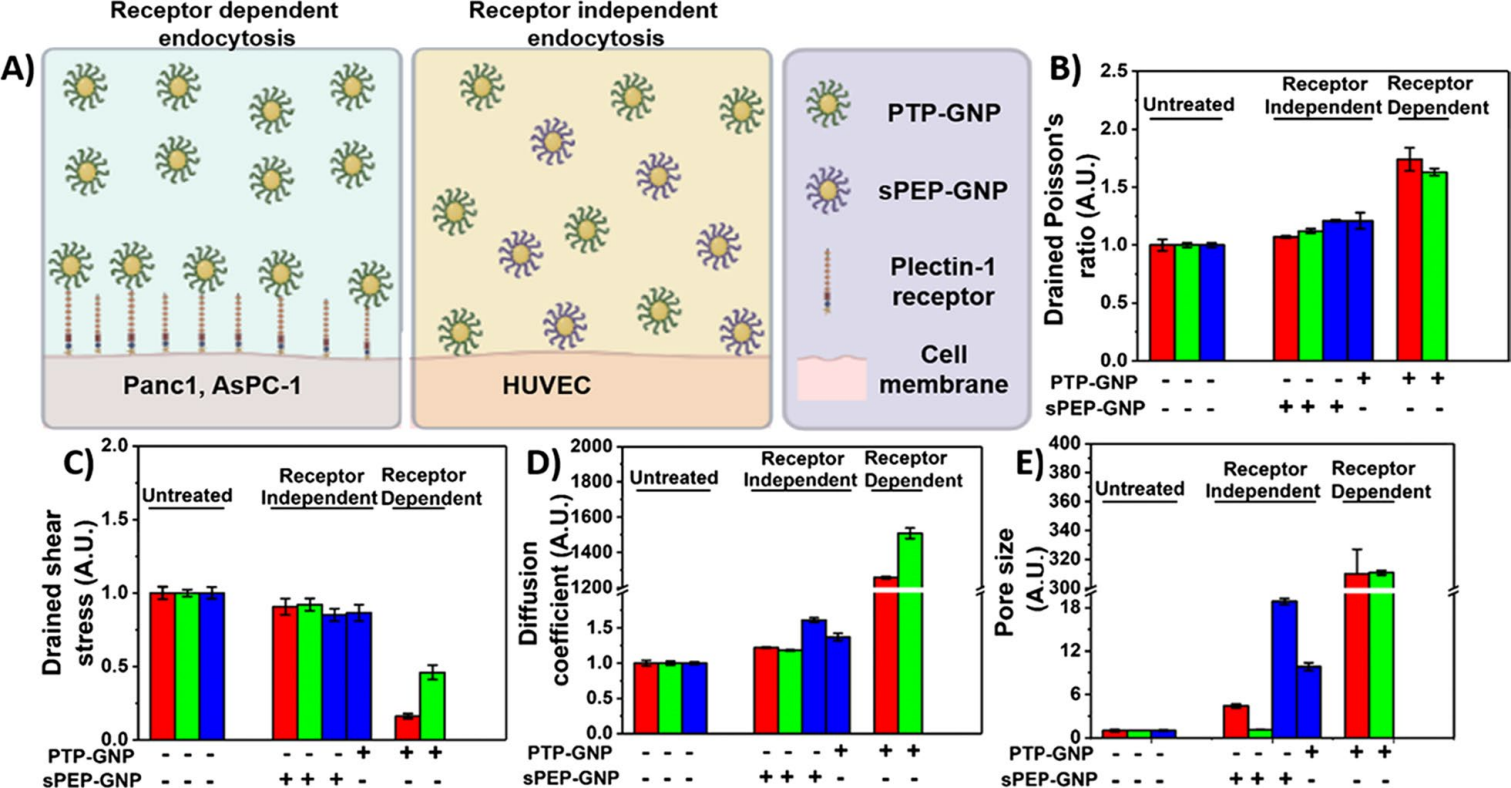
Comparative analysis of poroelasticity parameters influenced by receptor dependent and independent endocytosis processes. **A** Schematic of receptor dependent and receptor independent endocytosis process. Normalized poroelasticity parameters at the end of 20-min time window corresponding to **B** Drained shear stress. **C** Drained Poisson’s ratio. **D** Diffusion coefficient. **E** Pore size

Thus far, several studies have focused on the morphological alteration in cell membrane such as the pit formation and membrane ruffling during nanoparticle uptake process [54, 64]. Some prior studies including ours, focused on time-dependent alterations in membrane stiffness during receptor mediated endocytosis process [12, 62]. And while the biochemics involved in receptor dependent endocytosis process is well understood, alterations in poroelastic parameters during receptor dependent and independent endocytosis mechanisms have not been studied before. Here, using the AFM tool we study the dynamic alterations in poroelastic properties of cell membrane in Panc1 and AsPC-1 cells treated with PTP-GNP (receptor dependent) as well as Panc1 and AsPC-1 cells treated with sPEP-GNP, HUVECs treated with PTP- and sPEP-GNP (receptor independent) over a time window of 20 min, which was sufficient to capture the dynamic events as significant changes occurred during the initial 5 min time point and remained more or less constant at the end of 20-min time window. Plectin-1 protein, a novel biomarker for pancreatic cancer detection provides and maintains cellular mechanical integrity [54, 65]. As shown in our previous study [12], during receptor dependent endocytosis process, PTP-GNP binds to the membrane surface plectin-1 receptors and internalize, due to which there was a significant decrease in membrane plectin-1 expression as seen from the schematic Fig. 7A. Due to the absence of surface plectin-1 receptors in normal endothelial cells such as HUVECs, receptor independent mechanisms are more prevalent as shown in schematic Fig. 7A. With the loss in plectin-1, Panc1 cells appear significantly softer with time [12]. Herein, we attribute that the loss of mechanical integrity leads to opening/widening of pores that can allow the built-up interstitial pressure (due to rapid indentation) to dissipate rapidly thus, increasing diffusion coefficient and pore during receptor dependent mechanism.

During receptor independent endocytosis mechanism, the uptake of these GNPs might create significantly smaller pores through which, the built-up pressure is released. As the plectin-1 receptors, which maintain cellular mechanical integrity are not involved in this mechanism, we observed diffusion coefficient and the pore size to be significantly lesser compared to the receptor dependent endocytosis mechanism. We observed multifold increase in poroelastic parameters during receptor dependent endocytosis mechanism compared to receptor independent endocytosis as shown in Fig. 7B–E and attribute such a behavior to the presence of surface plectin-1 receptors, which has been shown to be present in Panc1 and AsPC-1 cells [12].

With the advent of technology, using AFM we can now evaluate nonlinear (poroelastic) properties such as drained Poisson’s ratio, diffusion coefficient and pore size of soft biological samples [66–71] in addition to the linear NMPs and serve several advantages over conventional techniques such as optical tweezers, magnetic tweezing cytometry and mechanical micropipette aspiration [63]. AFM’s unique capability of applying an external trigger force to study the response of the sample in a physiologically favorable condition at nanometer spatial resolution makes it a popular choice. As the indenter is rapidly approached and indented on to the cell membrane, an interstitial fluid pressure builds up within the cell giving rise to undrained condition. The redistribution of the interstitial fluid pressure (drained condition) is both time and length scale dependent and through force-relaxation, the effect of osmotic and cytoskeletal perturbations on cellular rheology can be understood with poroelasticity model [44]. Here, we observed a significant change in effective shear stress as the cell transitions from undrained to drained condition. It is essential to study the effect of external stimulus on cells as they are often subjected to fluid shear stress generated by the blood flow in the vascular microenvironment and interstitial flows in the tumor microenvironment [72]. In one of the studies using biomimetic microfluidic system, it was shown that the uptake of polystyrene nanoparticles in biomimetic dynamic conditions (cells are under higher shear stress than static system) by cancer cells was higher than that in a static system [72]. Similar study has been performed in different cell lines such as Human Embryonic Kidney (HEK) 293 T cells, Panc1 cells, human lung adenocarcinoma (A549) cells and human colorectal adenocarcinoma (HT29) cells. It was observed that in the presence of biomimetic shear stress, the cellular uptake of Doxorubicin was significantly higher than in static environment, which also affected its cell killing efficiency [73]. In our study, we observed a significant increase in effective change in shear stress when the cell transitions from undrained to drained state in the case of receptor mediated endocytosis mechanism. Our study supports the results mentioned in other studies and quantifies realtime alterations in effective shear stress during dynamic endocytosis process. Lastly, alterations in linear as well as nonlinear NMPs in biological cells rely heavily on actin filaments, microtubules as well as keratin intermediate filaments (KIF) [74]. Previously, a direct correlation between the structure of the KIF network and its local mechanical properties in alveolar epithelial cells was demonstrated [62]. In that study, shear stress applied across the cellular surface induced structural remodeling of the KIF due to mediation of the phosphorylation of K18pSer33, an essential protein involved in reorganization of the KIF network leading to increased cell membrane stiffness [74]. Our observations from the present and previously published study indicate a direct correlation between cell membrane stiffness and drained shear stress in receptor dependent endocytosis process [12].

We observed significant increase in diffusion coefficient and pore size during the receptor mediated endocytosis mechanism. Although, we observed a small increase in the diffusion coefficient and pore size during receptor independent mechanisms, they were approximately 1000-fold and 200-fold lesser, respectively than corresponding values observed during receptor dependent mechanisms as shown in Fig. 7D and E. While we observe multi-fold increase in diffusion coefficient, drained Poisson’s ratio and pore size; drained shear stress was observed to be following a decreasing trend contrary to the aforementioned poroelastic parameters. The fold-drop drained in shear stress in receptor dependent endocytosis was significantly more than receptor independent endocytosis as seen from Fig. 7C. Moreover, significant alterations in their values occurred during the initial 5-min time window after respective GNP-formulation treatment. From our previous study, we demonstrated a positive correlation between membrane stiffness and surface plectin-1 expression levels in Panc1 cells [12]. However, these fold changes in the membrane stiffness were several decades-fold lesser than the diffusion coefficient and pore size as observed in this study. Thus, these nonlinear poroelastic parameters prove to be more definitive indicators of the dynamic alterations in membrane dynamics.

Similar mechanism has been reported in HeLa S3 cells [44]. In this study, cytoskeletal component such as actin filaments and not the microtubules and keratin intermediate filaments played a key role in regulating cellular rheology [44]. In fact, the diffusion coefficient strongly depended on the actomyosin [44]. Moreover, depolymerizing the F-actin cytoskeleton decreased membrane stiffness, increased pore size and resulted in an overall increase in diffusion coefficient, a behavior similar to our observations [12, 44]. Similarly, in MDA-MB-231 cells, it was observed that with higher indenting velocities as the cells appeared stiffer, they were observed to be less poroelastic with lower diffusion coefficient and reduced pore size [50] coinciding with our results. And finally, poroelastic parameters of hepatocellular carcinoma (SMMC-7721) cells when subjected to fullerenol treatment were observed to be higher (both diffusion coefficient and pore size) with a decrease in cell membrane stiffness corroborating the relationship between membrane stiffness, diffusion coefficient and pore size similar to our observation [12, 49]. Above studies aid us in establishing a correlation in poroelastic parameters and cell membrane stiffness. To the best of our knowledge, our study is first of its kind to shed light into the nonlinear poroelastic alterations in membrane dynamics during receptor mediated endocytosis process. This will not only serve as key signatures to distinguish receptor dependent from independent endocytosis mechanism but will also aid us in improving our understanding of cellular rheology changes during various endocytosis mechanisms.

## Conclusion

Employing AFM’s ramp script technique that yielded F-R curves, poroelastic alterations in membrane dynamics of Panc1, AsPC-1 and HUVECs were evaluated. Permutation and combination of PTP-GNP and sPEP-GNP treatments in above cell lines gave rise to the receptor dependent and independent endocytosis mechanisms. The loss of plectin-1 receptor proteins at Panc1 and AsPC-1 cell membrane during receptor mediated endocytosis process opened/widened the pores that allowed the built-up interstitial pressure during rapid indentation to be released exhibiting a higher diffusion coefficient and pore size compared to receptor independent endocytosis mechanism. Poroelastic parameters proved to be more definitive indicators of the dynamic alterations in membrane dynamics compared to linear parameters during various endocytosis processes. We demonstrate that AFM is a promising tool to segregate receptor dependent from independent process based on poroelastic parameters.

## Experimental section

### Cell culture

Human pancreatic cancer cell line Panc1 and AsPC-1 were purchased from American Type Culture Collection (ATCC) and used with no further validations. Panc1 and AsPC-1 cells were cultured at ~ 60–70% confluency in separate 60 mm dishes with Gibco Dulbecco's Modified Eagle media (DMEM); supplemented with 10% Fetal Bovine Serum (FBS) and 1% Penicillin Streptomycin at 37 °C in a humidified 5% CO_2_ atmosphere. These cells were then arrested in S phase using 20 μg/mL aphidicolin for 12 h according to our previous study [75]. Prior to the experiments, culture media was replaced with Leibovitz-15 (L-15) media containing aphidicolin to continue arresting cells in S phase during the course of the experiments. Human umbilical vein endothelial cells (HUVECs) purchased from Lonza, were cultured at 60–70% confluency in a 60 mm dish with on plates coated with 30 mg/mL collagen type 1 and cultured in endothelial basal medium (EBM) supplemented with EBM-MV Bullet Kit (5% fetal bovine serum in EC basic medium with 12 µg/mL bovine brain extract, 1 µg/mL hydrocortisone, 1 µg/mL GA-1000). HUVECs were serum starved (0.25% fetal bovine serum) for 6 h prior to the experiment [76]. Just before the experiment, EBM was replaced with L-15 media with 2% FBS to continue to arrest HUVECs. Both Panc1 and HUVECs were arrested to overcome the heterogeneity in their NMPs arising from cells in various phases.

### Synthesis of gold nanoparticles

Synthesis of gold nanoparticles conjugated with plectin-1 targeted peptide (PTP-) or scrambled peptide (sPEP-) has been described in our previous studies (published in Nanoscale) [4, 12]. Briefly, Gold (III) chloride trihydrate and peptide were dissolved in Milli-Q water in 10:1 molar ratio under continuous stirring for 2 min at 37 °C. Then, 1 M NaOH was added drop by drop until the overall pH of the solution was achieved at 12 and further subjected to continuous stirring for 16 h. GNPs were isolated by ultracentrifugation at 38 K RPM using Beckmann Optima L-80 XP ultracentrifuge (purchased from Beckman Coulter Inc.). Supernatant was removed carefully to yield pellet of PTP-GNP or sPEP-GNP sedimented at the bottom of the centrifuge tube. Finally, Milli-Q water was added to the collected pellet to make the final volume same as the reaction mixture. For experiment purpose, above solutions were further diluted tenfold to constitute a working stock solution and stored at 4 °C until further use. A comprehensive characterization of these gold nanoparticle formulations have been performed in our previous study [4], where the overall size of PTP-GNP and sPEP-GNP was ~ 5 nm and the addition of external surface moieties did not significantly alter their dimensions.

### Atomic force microscopy

Dimension Fast Scan with Scanasyst (Bruker Corp.) AFM was employed with LC CAL A probes to perform ramp script analysis mode that yielded force-relaxation (F-R) curves on both Panc1 and HUVECs in fluid environment to preserve their mechanical integrity. 60 mm culture dishes consisting of Panc1 or HUVECs were held on to the AFM stage using a custom-built plate holder. Due to the overall heterogeneity in cell membrane, we chose a 500 × 50 nm^2^ region over the nucleus for all the AFM studies [75]. Bruker’s MIRO View allowed us to maintain the location of these data points to be constant throughout the dynamic studies over a period of 20 min. This spherical LC-CAL A probe bears a nominal spring constant of 0.1 N/m and tip radius of 70 nm. It is made of silicon tip on nitride lever with a resonant frequency of 45 kHz. The length of the cantilever corresponds to 54 µm, which along with the spring constant and tip radius makes it an ideal candidate to study biological cells. Following the laser alignment on the reflective side of the tip to yield maximum signal strength, it was further calibrated in Milli-Q water to determine the spring constant and deflection sensitivity of 0.06 N/m and 55.7 nm/V, respectively. Moreover, the spherical surface area in contact with the cell membrane allows for optimal contact without creating any permanent fractures in the cell membrane. Then we optimized the Quantitative Nanomechanical Mapping (QNM) sync distance for the probe at 1 kHz frequency by measuring its deflection sensitivity to be within ± 5% range of the tip's deflection sensitivity measured via thermal calibration for accurate tip-sample interaction. Experimental approach adopted to study the poroelasticity parameters was force-relaxation (F-R). F-R allows monitoring the relaxation in the force at a constant height (indentation). Our sample set comprised of 7 cells: each cell with 7 data points over a 20-min time window in the increments of 5 min. All the experiments were performed at 37° C maintained using a temperature-controlled AFM stage.

### Poroelasticity model theory

The detailed explanation of the theoretical model of poroelasticity and its application is well described in the previous studies [77, 78]. Poroelasticity model yields quantitative parameters such as the drained Poisson’s ratio (υ_d_), drained shear stress (G’’), the diffusion coefficient (D_p_) and the pore size (κ). For nanoindentation process on soft samples such as the cells, probes with spherical tip geometry have been commonly employed to yield an accurate force-distance curve and meet the deformation criteria without causing plastic deformation the cells. Hence, the Hertzian model is commonly employed on the retrace curve of the force distance curve and the model is as follows,

1 $$F = \left( \frac{8}{3} \right)\left( {\frac{E}{{1 - \vartheta_{u} }}} \right)R^{\frac{1}{2}} \delta^{\frac{3}{2}}$$

where, F is the force on the tip, E is the Young’s modulus, $\vartheta_{u}$ is undrained the Poisson’s ratio, R is the radius of the indenter and δ is the indentation. By fitting the retrace curve to yield maximum linearity coefficient value, E can be obtained. Further, elastic modulus and shear modulus (G) are linearly related through the Poisson’s ratio.

2 $$E = 2G\left( {1 + \vartheta_{u} } \right)$$

In the force-relaxation experiment, the cantilever is approached at high forward velocity due to which the cytoplasmic interstitial fluid does not have sufficient time to drain out of the compressed region. This is identified as the undrained scenario for poroelastic material [77, 79]. The Eq. 1 becomes,

3 $$F\left( 0 \right) = \left( \frac{16}{3} \right)G^{\prime}R^{\frac{1}{2}} \delta^{\frac{3}{2}}$$

As the force reaches a plateau, the interstitial fluid redistributes in the cell and the force inflicted by the indenter is balanced by the stress in the elastic porous matrix only. This condition is termed as the drained condition and Eq. 1 becomes,

4 $$F\left( \infty \right) = \left( \frac{8}{3} \right)\left( {\frac{1}{{1 - \vartheta_{d} }}} \right)G^{\prime\prime}R^{\frac{1}{2}} \delta^{\frac{3}{2}}$$

where, $\vartheta_{d}$ is the drained Poisson’s ratio.

Comparing Eqs. 3 and 4 yields,

5 $$\left( {\frac{F\left( 0 \right)}{{F\left( \infty \right)}}} \right) = 2\left( {1 - \vartheta_{d} } \right)$$

F (0) and F (∞) can be extracted from the force-relaxation curve. Equation 5 yields drained Poisson’s ratio. By substituting $\vartheta_{d}$ in Eq. 4, we can obtain drained shear stress (G’’). Moreover, Eqs. 3 yields undrained shear stress (G’) as F(0), R and $\vartheta_{d} )$ are known quantities. There fails to exist any closed form analytical solution for indentation of a poroelastic infinite half-space by a spherical indenter exists. However, an approximate solution is obtained by finite- element simulation [80] given by,

6 $$\frac{F\left( t \right) - F\left( \infty \right)}{{F\left( 0 \right) - F\left( \infty \right)}} = 0.491e^{ - 0.908\sqrt \tau } + 0.509e^{ - 1.679\tau }$$

where, τ is the characteristic poroelastic time required for the force to relax. F(t), F(0) and F(∞) are all extracted from the force relaxation curve. The diffusion constant (D_p_) of the porous membrane is related to the characteristic time by;

7 $$\tau = \left( {\frac{{D_{p} t}}{R}} \right)$$

Diffusion coefficient is further directly linked to the pore size given by,

8 $$D_{p} = \frac{{2G^{\prime\prime}\left( {1 - \vartheta_{d} } \right)}}{{1 - 2\vartheta_{d} }}\kappa$$

where, $\kappa$ is the pore size. Equations 4, 5, 7 and 8 cumulatively yield the poroelasticity parameters such as G’’, $_{d}$, $D_{p}$ and $\kappa$, respectively.

### Data analysis of AFM experiments

Bruker’s Nanoscope analysis v1.9 was used to extract values corresponding to the F-R curves, which were then, analyzed using a custom-built MATLAB programming to yield values corresponding to various poroelastic parameters. Origin Pro Lab software was then used for statistical analysis and graphical presentation. After ensuring that these datasets satisfy normality criteria, One Way ANNOVA was performed to calculate the significance between the datasets.

## Supporting information

Supporting information comprises of five figures that support the main findings mentioned in the manuscript. These figures comprise of representative force-relaxation curves for Panc1, AsPC-1 and HUVECs under no treatment. Baseline experiments exhibiting temporal alterations in poroelasticity parameters S phase Panc1 cells in the presence of the AFM tip applied stimulus, alterations in synchronous and asynchronous AsPC-1 cells in the presence of the AFM tip applied stimulus, comparative analysis of poroelasticity parameters in synchronous Panc1 and HUVECs without any treatment and lastly, alterations in drained shear stress during receptor dependent and independent endocytosis process.

## Supplementary Information

**Additional file 1: Figure S1**. Representative relaxation segments from force-relaxation curves for different cell lines with no treatment. A) Panc1. B) HUVEC. C) AsPC-1. **Figure S2**. Temporal alterations in poroelasticity parameters in untreated S phase synchronized Panc1 cells under an external stimulus of 500 pN. A) Drained Poisson’s ratio. B) Diffusion coefficient. C) Pore size. **Figure S3**. Temporal alterations in poroelasticity parameters in untreated S phase synchronized as well as asynchronized AsPC-1 cells under an external stimulus of 500 pN. A) Drained Poisson’s ratio. B) Diffusion coefficient. C) Pore size. **Figure S4**. Comparative analysis of poroelasticity parameters in untreated Panc1 and HUVECs. A) Drained modulus. B) Diffusion coefficient. C) Pore size. (Statistical significance calculated using One Way ANNOVA. ns, not significant; ***,p<0.001; ****,p<0.0001). **Figure S5**. Temporal alteration in drained shear stress. A) In Panc1 cells during receptor dependent (PTP-GNP in Panc1) and receptor independent (sPEP-GNP in Panc1) endocytosis processes. B) In AsPC-1 cells during receptor dependent (PTP-GNP in AsPC-1) and receptor independent (sPEP-GNP in AsPC-1) endocytosis processes. C) In HUVECs with PTP-GNP and sPEP-GNP treatment both resembling receptor independent endocytosis process.

## Data Availability

The datasets used and/or analyzed during the current study are available from the corresponding author on reasonable request.
