# Supplementary material for: Dynamic alteration of poroelastic attributes as determinant membrane nanorheology for endocytosis of organ specific targeted gold nanoparticles
Source: J Nanobiotechnology. 2022 Feb 8;20:74. doi: 10.1186/s12951-022-01276-1 (PMC8822666; doi:10.1186/s12951-022-01276-1)
Supplement: Supplementary file 1 — Additional file 1: Figure S1. Representative relaxation segments from force-relaxation curves for different cell lines with no treatment. A) Panc1. B) HUVEC. C) AsPC-1. Figure S2. Temporal alterations in poroelasticity parameters in untreated S phase synchronized Panc1 cells under an external stimulus of 500 pN. A) Drained Poisson’s ratio. B) Diffusion coefficient. C) Pore size. Figure S3. Temporal alterations in poroelasticity parameters in untreated S phase synchronized as well as asynchronized AsPC-1 cells under an external stimulus of 500 pN. A) Drained Poisson’s ratio. B) Diffusion coefficient. C) Pore size. Figure S4. Comparative analysis of poroelasticity parameters in untreated Panc1 and HUVECs. A) Drained modulus. B) Diffusion coefficient. C) Pore size. (Statistical significance calculated using One Way ANNOVA. ns, not significant; ***,p<0.001; ****,p<0.0001). Figure S5. Temporal alteration in drained shear stress. A) In Panc1 cells during receptor dependent (PTP-GNP in Panc1) and receptor independent (sPEP-GNP in Panc1) endocytosis processes. B) In AsPC-1 cells during receptor dependent (PTP-GNP in AsPC-1) and receptor independent (sPEP-GNP in AsPC-1) endocytosis processes. C) In HUVECs with PTP-GNP and sPEP-GNP treatment both resembling receptor independent endocytosis process. [file 12951_2022_1276_MOESM1_ESM.docx]

**Dynamic Alteration of Poroelastic Attributes as Determinant Membrane Nanorheology for Endocytosis of Organ Specific Targeted Gold Nanoparticles**

Tanmay Kulkarni†, Debabrata Mukhopadhyay†,‡, Santanu Bhattacharya†,‡*

†Department of Biochemistry and Molecular Biology, Mayo College of Medicine and Science, Jacksonville, FL, USA.

‡Department of Physiology and Biomedical Engineering, Mayo College of Medicine and Science, Jacksonville, FL, USA.

*Address Correspondence to: Santanu Bhattacharya, Dept. of Biochemistry and Molecular Biology, Griffin 413, Mayo Clinic Florida, 4500 San Pablo Road S, Jacksonville, FL-32224, Tel: (904) 953-0507; Fax: (904) 953-0277;

E-mail: [bhattacharya.santanu@mayo.edu](mailto:bhattacharya.santanu@mayo.edu)

**Supplementary figures:**

**Figure S1**

**
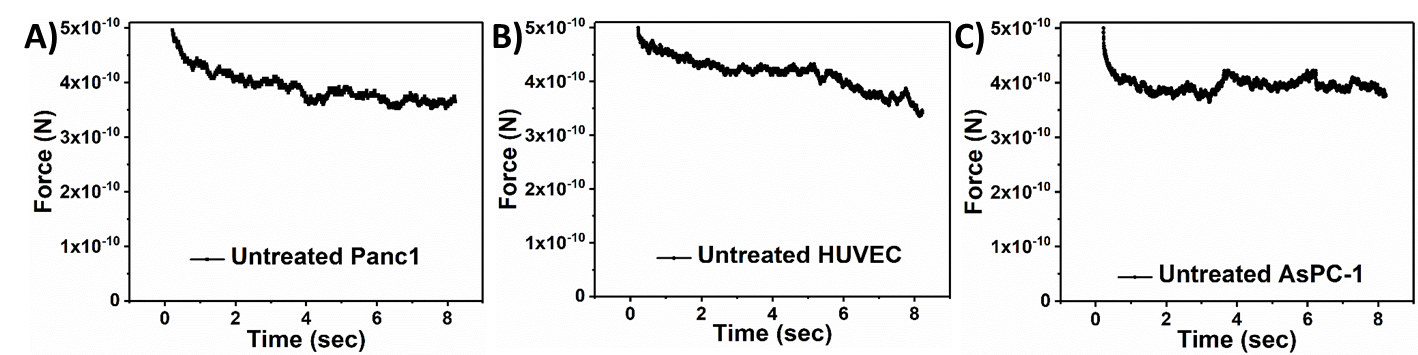
**

**Figure S1. Representative relaxation segments from force-relaxation curves for different cell lines with no treatment.** A) Panc1. B) HUVEC. C) AsPC-1.

**Figure S2**

**Figure S2. Temporal alterations in poroelasticity parameters in untreated S phase synchronized Panc1 cells under an external stimulus of 500 pN.** A) Drained Poisson’s ratio. B) Diffusion coefficient. C) Pore size.

**Figure S3**

**Figure S3. Temporal alterations in poroelasticity parameters in untreated S phase synchronized as well as asynchronized AsPC-1 cells under an external stimulus of 500 pN.** A) Drained Poisson’s ratio. B) Diffusion coefficient. C) Pore size.

**Figure S4**

**Figure S4. Comparative analysis of poroelasticity parameters in untreated Panc1 and HUVECs.** A) Drained modulus. B) Diffusion coefficient. C) Pore size. (Statistical significance calculated using One Way ANNOVA. ns, not significant; ***,p<0.001; ****,p<0.0001).

**Figure S5**

**Figure S5. Temporal alteration in drained shear stress.** A) In Panc1 cells during receptor dependent (PTP-GNP in Panc1) and receptor independent (sPEP-GNP in Panc1) endocytosis processes. B) In AsPC-1 cells during receptor dependent (PTP-GNP in AsPC-1) and receptor independent (sPEP-GNP in AsPC-1) endocytosis processes. C) In HUVECs with PTP-GNP and sPEP-GNP treatment both resembling receptor independent endocytosis process.
